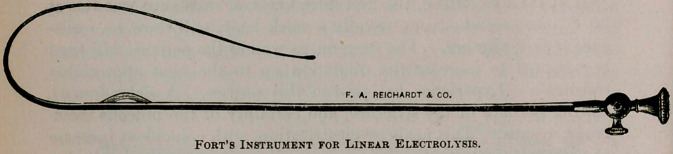# The Non-Operative Treatment of Urethral Strictures*Read before the New York County Medical Society, May 25, 1896.

**Published:** 1896-10

**Authors:** Fred C. Valentine

**Affiliations:** New York; Genito-Urinary Surgeon, West Side German Dispensary, etc., etc.


ATLANTA

Medical and Surgical Journal.

VOL. XIII. OCTOBER, 1896. No. 8.

LUTHER B. GRANDY, M.D., MANAGING EDITOR.

M. B. HUTCHINS, M.D., BUSINESS MANAGER.

ALEX. W. STIRLING, M.D., C.M., M.B. (Edin.), D.P.H. (Lond.), MANAGING EDITOR PRO TEM.

COLLABORATORS:

A. W. CALHOUN, M.D., LL.D., VIRGIL O. HARDON, M.D., FLOYD W. McRAE, M.D., AND DUNBAR ROY, M.D.

### ORIGINAL COMMUNICATIONS.

### THE NON-OPERATIVE TREATMENT OF URETHRAL STRICTURES.*

By FRED C. VALENTINE, M.D.,

NEW YORK.

(Genito-Urinary Surgeon, West Side German Dispensary, etc., etc.)

It will, doubtless, appear at once, that the title of this paper is essentially a misnomer, as every instrumental invasion of the urethra is necessarily a surgical operation. It being such, too much care cannot be bestowed upon its details.

This paper, being intended for general practitioners, will be guided from the more intricate and abstruse special considerations.

In the very onset, to be just to the principles that in practice have stood the test of time, indiscriminate cutting of strictures must be emphatically reprobated. Growing experience gives daily conviction that the non-operative, or better, "bloodless" method so ably worked out by Oberlaender and his followers, principally Kollmann

*Read before the New York County Medical Society, May 25, 1896.

of Leipzig, Wossidlo of Dresden, and others, is the really correct manner of treating the vast majority of strictures.

While desiring strictly to limit myself now to the question of treatment, I am obliged to touch very lightly upon a few other elements, to make clear the rationale of the subject.

*Etiology.*—Posner [1] says: "The development of cicatricial tissue and the resultant contraction of the urethral canal, plays a most important part among the sequelae which may attend chronic inflammation of the lower urinary tract." As this development of cicatricial tissue proceeds imperceptibly, it is evident that its treatment must be associated with the treatment of its cause.

*Prophylaxis.*—Accepting the above etiology (we are not now considering traumatisms, malignant or benign neoplasms, or any other cause), the prevention of stricture manifestly lies in rational treatment of urethral inflammations, acute and chronic. *(Posner, ibid.)*

*Classification.*—For the present purposes it will suffice to grossly classify the subject, into soft strictures, most developed hard infiltrates (Oberlaender), and spasmodic strictures. The more correct, detailed divisions would be in place in a paper for genito-urinary specialists.

We may, for the present, call strictures soft, at the initial stages of their development; most developed hard infiltrates, as their name conveys. They are the extremes, as far as concerns the constitution of strictures.

Spasmodic strictures are differently viewed by the authorities. Some entirely deny their existence; among them Posner *(ibid.)* says: "Spasmodic strictures, erstwhile often assumed, consist partly in the imagination of unskilled physicians and partly belong into the domain of the neuroses." It would seem that while neurotic strictures last, they are strictures to all intents.

Otis [2] says: "The presence of a contracted meatus urinarius or a stricture of large caliber, often unnoticed, is capable of exciting chronic spasmodic closure of the membranous urethra, quite indistinguishable from true organic stricture. . . . ."

Wossidlo [3] generalizes as follows: "Stricture conveys to me the impassability of any part of the urethra by a bougie which penetrates the meatus smoothly."

---

[1] "Therapie der Harnkrankheiten," Berlin, 1895.
[2] Stricture of the Male Urethra, 1880 (page 302).
[3] Zur Dilatationsbehandlung der Harnrohren-Stricturen.—*Berliner klinische Wochenshrift,* No. 6, 1896.

This is the practical aspect with which we have to deal.

The symptoms and course of urethral stricture, if here discussed, would lead far beyond the confines of a paper on treatment.

*Diagnosis.*—The vast majority of strictures can be located and measured successfully only by the soft bougie-a-boule. The form I prefer is that with a rather abrupt shoulder, which catches readily in the stricture, as it is drawn out of the urethra. I have, however, seen some very fine strictures, which were so soft and of such large caliber that they did not become apparent except by the urethroscope.

*Treatment.*—Wossidlo *(ibid.)* sums up the current treatment of strictures with sounds as follows:

" . . . Elastic bougies are used, increasing one number at each sitting. . . . When 18 or 29 F. is reached, metal sounds replace the rubber bougies. . . . It is generally deemed sufficient when the number is reached that will pass a not abnormally contracted meatus. Most cases are considered cured when 23 to 25 F. can be easily introduced. They are then discharged, with the advice to prevent recontraction by passing sounds."

But this is by no means curing a stricture. As I said before, strictures have come under my observation, that were revealed only by the urethroscope. If unnoticed, they would have followed nature's rule and contracted, imperceptibly perhaps, until much suffering and great danger became inevitable.

Manifestly, then, the only true scientific guide to the diagnosis, treatment and final discharge of a case of urethral stricture, is the urethroscope.

As soon as a stricture is sufficiently dilated to allow an adequately wide tube to enter it, the diseased region should be examined with the urethroscope. Many cases which permit 24 to 25 F. to pass easily and are discharged as cured, by no means show a healthy urethra.

It is just this readiness to dismiss cases when all discharge and other symptoms have ceased, that causes so much havoc. I have discussed this in a paper on the marriage of gonorrheal patients. [1]

The need of a better understanding of stricture and its congener, chronic urethritis, is made painfully manifest through a case which recently came under the observation of my assitant, Dr. W. E. de Salazar. The patient, who had been treated for gonorrhea by a

1 When May Gonorrheal Patients Marry ?—*American Medico-Surgical Bulletin*, Oct. 1, 1896.

general practitioner of more than average good repute, complained of a thick, greenish-yellow morning drop. His medical adviser told him that the drop was of pathological import equal to the mucus he hawked from his throat every morning!

If a good practitioner will treat the morning drop so lightly, it is small wonder that so little heed is paid by patients to shreds, flakes, filaments and granules in the urine. But all this shows the need of a better understanding of the treatment of stricture, one of the causes of the above manifestations.

An outline of the "bloodless" treatment of stricture may now be offered.

Those strictures which barely permit the passage of a filiform bougie, naturally tempt one to think of external urethrotomy. But it is remarkable how rapidly most of such extreme narrowings will yield to the fine sounds (Gouley's or Banks'). Until these can be followed by a small rubber catheter, it may be necessary to relieve the bladder by tapping or aspirating through Retzius's space.

Once that a fine catheter has entered, it should be fastened there and plugged, with orders to evacuate every 3 or 4 hours or oftener, according to the conditions of the urine. Twenty-four hours later the catheter will be found lying loosely in the urethra, and may be easily replaced by one a number or two larger. I have distinctly in mind a case treated by Wossidlo, in Berlin, in which I assisted him. In nine days' time four filiform strictures so yielded that they easily admitted a 20 F.

When, however, 17 F. is reached, the case may be well managed with metal sounds. As regards the shape thereof, I always use that designated by Benique. All physicians who have used them, and patients who have had them introduced, will prefer them to all other forms. Lying as they do in the physiological curve of the urethra, they exercise dilatation without traction upon the canal.

OBERLAENDER DILATOR FOR THE ANTERIOR URETHRA.

When, however, the limit is reached, which will pass the normal meatus smoothly, we must either slit it or proceed to other means of dilatation. Slitting the meatus, simple as it is, will oftentimes fail us, principally because we cannot, without making a gross hypospadias, slit it large enough to permit passing a sound sufficiently large to exercise the requisite amount of pressure upon the contracture. Thus it will be well, before proceeding to this mutilation, to give heed to the teachings of Oberlaender.

Oberlaender's dilators, as well as Kollmann's, give us an armamentarium which, if carefully employed, enable us to cure perhaps 99 per cent. of all strictures. I say this advisedly, mainly as the result of not a small personal experience.

The method of using these dilators is exceedingly simple and, when properly executed, need not give the patient any pain. For convenience and other reasons, I have slightly modified the technique of their employment.

Holding the closed dilators in the right hand, the point is made to dip up as much powdered talcum as possible. Then the rubber cover, previously rendered aseptic by scrubbing and washing in a solution of creolin or lysol and dried, is slipped over the dilator.

The rubber cover then being lubricated it is carefully passed through the stricture and dilatation very gradually begun. When the first slight resistance is felt, the dilator is left in the urethra for five minutes. Then an intravesical irrigation without a catheter is made my means of the apparatus devised for the purpose. [1]

OBERLAENDER DILATOR FOR THE ANTERIOR URETHRA AND BULBOUS PORTION.[2]

If at the first sitting, the first intra-urethral resistance was felt at 20 F., the second sitting, usually a week later, will show no resistance at that number. The surgeon, as well as the patient, will then be tempted to increase the dilatation up to the next appreciable resistance. Experience has proven this unwise. A *debridement*, tearing perhaps of the stricture, and certainly of the mucous membrane, results. This heals by cicatrization, with coincident increase of urethral contraction. It is never well, except perhaps in the beginning, to increase over one number at each sitting, and then never over two numbers.

Later on, when the urethroscope shows that the stricture is getting markedly less, the weekly intervals may be increased to ten days, two weeks, and gradually to four weeks. As the intervals increase, the increase of each dilatation should be reduced to half a

1 Technique of Urethral and Intravesical Irrigations.—*Clinical Recorder*, February, 1896.
2 Kollmann's four-branched dilators were depicted in the *Clinical Recorder* for April, 1896.

number or even less, but each sitting prolonged. The longest sitting that can be borne with comfort, even in hot weather, is 45 minutes. It will then be found that a dilatation which made the urethra fit quite snugly on the dilator will make it lie very loosely about the instrument.

In the beginning of treatment each dilatation is followed by quite a copious discharge of mucus, pus, and epithelium, with bacteria. At least, this is the experience of others and was mine, until I followed each sound and each dilator with an intravesical irrigation of potassic permanganate, 1 to 6,000, or of boric acid, 2 per cent. Since then, now fourteen months, few of my patients, private or dispensary, have had any more than a very slight discharge, and most of them none at all, after ingression of any kind into the urethra.

In cases which previously had "catheter-fever" I wash out the urethra with the same apparatus, and having ascertained the capacity of the bladder, I fill it one-third or one-half with either of the above solutions. Leaving the fluid in the bladder, while the dilator is used and washing afterwards in the same manner, has never been followed by "catheter-fever," even in the most susceptible cases.

I formerly followed Wossidlo and others in cocainizing the urethra before instrumentation. Careful observation, however, has led me to desist therefrom, as the patients complained of considerable pain for hours after the anesthesia had worn off. I now use cocaine only in cases of urethral hyperesthesia. This soon yields; then I abstain from it altogether.

FORT'S INSTRUMENT FOR LINEAR ELECTROLYSIS.

Recently Prof. Fort, of Paris, demonstrated his method of linear electrolysis in very deep and tight strictures, at Bellevue Hospital and other places. Prof. R. W. Taylor speaks in no scant praise of Fort's method. My observations on its use are limited, but shall be the subject of a future paper. If my success equals that of others, I shall certainly use it before beginning dilatations, with a view to gaining time.

But slight mention has been made of the treatment of acute retention of urine, due to stricture. Hot sitz-baths and opium will often render tapping or aspiration unnecessary, and where many efforts at passing a catheter have failed, the next endeavor is frequently followed with surprising success.

True, there are cases in which the emergency demands urethrotomy; but they are so exceedingly few in number, that they may safely be called exceptional.

In this connection Taylor [1] says: "The trend of thought as regards the treatment of urethral stricture of late years has been so unswervingly toward cutting operations, that many surgeons are wholly unaware of the beneficent and lasting effects of gradual dilatation. I have many times been pleasantly chaffed, and even mildly derided, about my conservative views as to the treatment of the male urethra when the seat of contractions; but after a not inconsiderable experience, stretching over a period of twenty-seven years, I am to-day more than ever convinced that cutting operations should be a last resort, and that intemperate incisions and overstretching are very frequently the cause of never-ending suffering and inconveniences."

The advocacy of Oberlaender, Kollmann, Wossidlo and many others, indorsed by so eminent an American authority, takes what may appear like temerity, from my striving to stay the way of the knife into the urethra.

[1] Pathology and Treatment of Venereal Diseases, 1895.

## Figures and Tables

**Figure f1:**
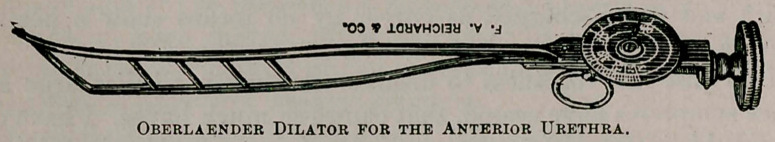


**Figure f2:**
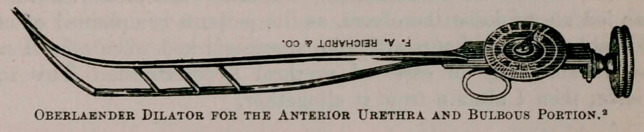


**Figure f3:**